# Leptin promotes bone metastasis of breast cancer by activating the SDF-1/CXCR4 axis

**DOI:** 10.18632/aging.103599

**Published:** 2020-08-18

**Authors:** Lixia Duan, Yongkui Lu, Weimin Xie, Li Nong, Yuxian Jia, Aihua Tan, Yan Liu

**Affiliations:** 1 The Fifth Department of Chemotherapy, Affiliated Tumor Hospital of Guangxi Medical University, Nanning, China; 2 Department of Trauma Orthopedic and Hand Surgery, The First Affiliated Hospital of Guangxi Medical University, Nanning, China; 3 Guangxi Key Laboratory of Regenerative Medicine, Guangxi Medical University, Nanning, China

**Keywords:** leptin, breast cancer, bone metastasis, SDF-1/CXCR4 axis

## Abstract

Obesity is associated with an increased risk of tumorigenesis, and increased leptin levels can promote tumor metastasis. However, the effects of leptin on bone metastasis in breast cancer are not fully understood. Here, we examined leptin receptor expression and bone metastasis in tissue samples from 96 breast cancer patients. In addition, we investigated the effects of leptin on the metastatic capacity of breast cancer cells *in* *vitro* using a transwell assays. The results indicated that higher leptin receptor levels in breast cancer cells are associated with increased incidence of bone metastasis in breast cancer patients. Additionally, leptin promoted migration and invasion of breast cancer cells. The SDF-1/CXCR4 axis activated by leptin also promoted bone metastasis of breast cancer. Finally, increased CXCR4 expression was accompanied by high leptin receptor expression in bone metastatic tissues from breast cancer patients. These results indicate that leptin induces bone metastasis of breast cancer by activating the SDF-1/CXCR4 axis.

## INTRODUCTION

Breast cancer is one of the most common cancers worldwide, and distant metastasis is a primary driver of poor prognosis in breast cancer patients [1, 2]. Bone tissue is the most common target of metastasis, and bone metastasis is observed in approximately 83% of advanced stage breast cancer patients. Bone metastasis can lead to a series of skeletal consequences such as pathologic fractures, pain, and nerve-compression syndromes [3, 4].

Obesity is a significant risk factor and negative prognostic factor for breast cancer, and the adipocyte-derived adipokine leptin links obesity and breast cancer [5, 6]. Leptin acts through specific leptin receptors (ObRs) and plays a key role in energy metabolism, food intake, and endocrine systems, among others. It has been reported that higher leptin expression is associated with increased tumor metastasis and poorer prognosis [7–9]. However, the underlying mechanisms linking leptin and metastasis remain poorly understood.

Metastasis is a complex process, and many factors, including properties of the tumor cells and of the local microenvironment in the target area, can influence tumor dissemination [10]. The chemokine SDF-1 is often produced at high levels by metastatic target organs [11]. CXCR4, the sole receptor for SDF-1, belongs to the heptahelical G protein-coupled receptor superfamily [12]. In breast cancer, organ-specific metastasis is thought to be associated with CXCR4 expression and downstream signalling [13, 14]. It has been reported that increased CXCR4 expression in breast cancer tissues is associated with the occurrence of tumor distant metastasis and decreased overall survival in patients [15]. Furthermore, CXCR4 upregulation in breast cancer cells enhanced the migratory potential of tumor cells towards SDF-1 producing organs [16]. In this study, we explored the effects of leptin on bone metastasis in breast cancer cells and underlying mechanisms.

## RESULTS

### Leptin receptor expression was associated with malignancy and bone metastasis in breast cancer patients

**Figure 1:**
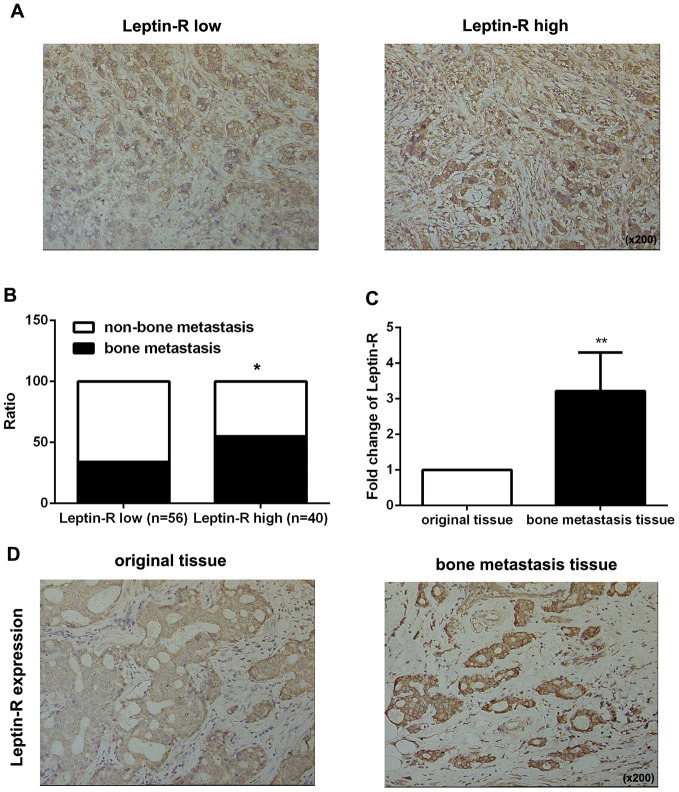
**Leptin receptor expression was associated with malignancy and bone metastasis in breast cancer patients.** (**A**) IHC staining was employed to examine leptin receptor expression in breast cancer tissues. (**B**) Frequencies of bone metastasis and non-bone metastasis in patients with low or high leptin receptor expression. (**C**) Realtime-PCR was used to measure leptin receptor expression in primary breast cancer tissue and bone metastasis tissue. The data are shown as means ± SD, **P<0.01. (**D**) IHC staining was employed to examine leptin receptor expression in primary breast cancer tissue and bone metastasis tissue.

First, we examined leptin receptor expression in 96 breast cancer tissues using IHC staining and identified correlations between leptin receptor expression and clinicopathologic features. As shown in Figure 1A, 56 patients had high leptin receptor expression and 40 had low leptin receptor expression. Statistical analysis revealed that high leptin receptor expression was significantly associated with occurrence of bone metastasis (Figure 1B and Table 1) and TNM stage compared to patients with low leptin receptor expression (Table 1).

**Table 1:** Immunohistochemical stain results and clinicopathologic features of 96 breast cancer patients.

**Clinicopathologic features**	**Leptin receptor expression**		**p value**
	**low**	**high**	
**Frequency (%)**	56 (58.33%)	40 (41.67%)	
**Age (years, mean±SD)**	42.14±9.737	50.34±11.531	
**T-stage (T1 vs >T1)**			
**T1**	38	16	**0.007
**>T1**	18	24	
**N-stage (N0 vs N+)**			
**N0**	34	18	0.127
**N+**	22	22	
**Grade (1, 2 vs 3)**			
**1, 2**	32	19	0.351
**3**	24	21	
**bone metastasis (%)**	19 (33.9%)	22 (55.0%)	*0.039

* P<0.05; **P<0.01.

Next, we examined expression of the leptin receptor in breast cancer-derived bone metastatic tissues. As shown in Figure 1C and 1D, leptin receptor expression was higher in metastatic tissues than in the original breast cancer tissues. Taken together, these results indicate that the leptin receptor promotes bone metastasis in breast cancer patients.

### Leptin promoted breast cancer cell metastasis and leptin receptor expression

**Figure 2:**
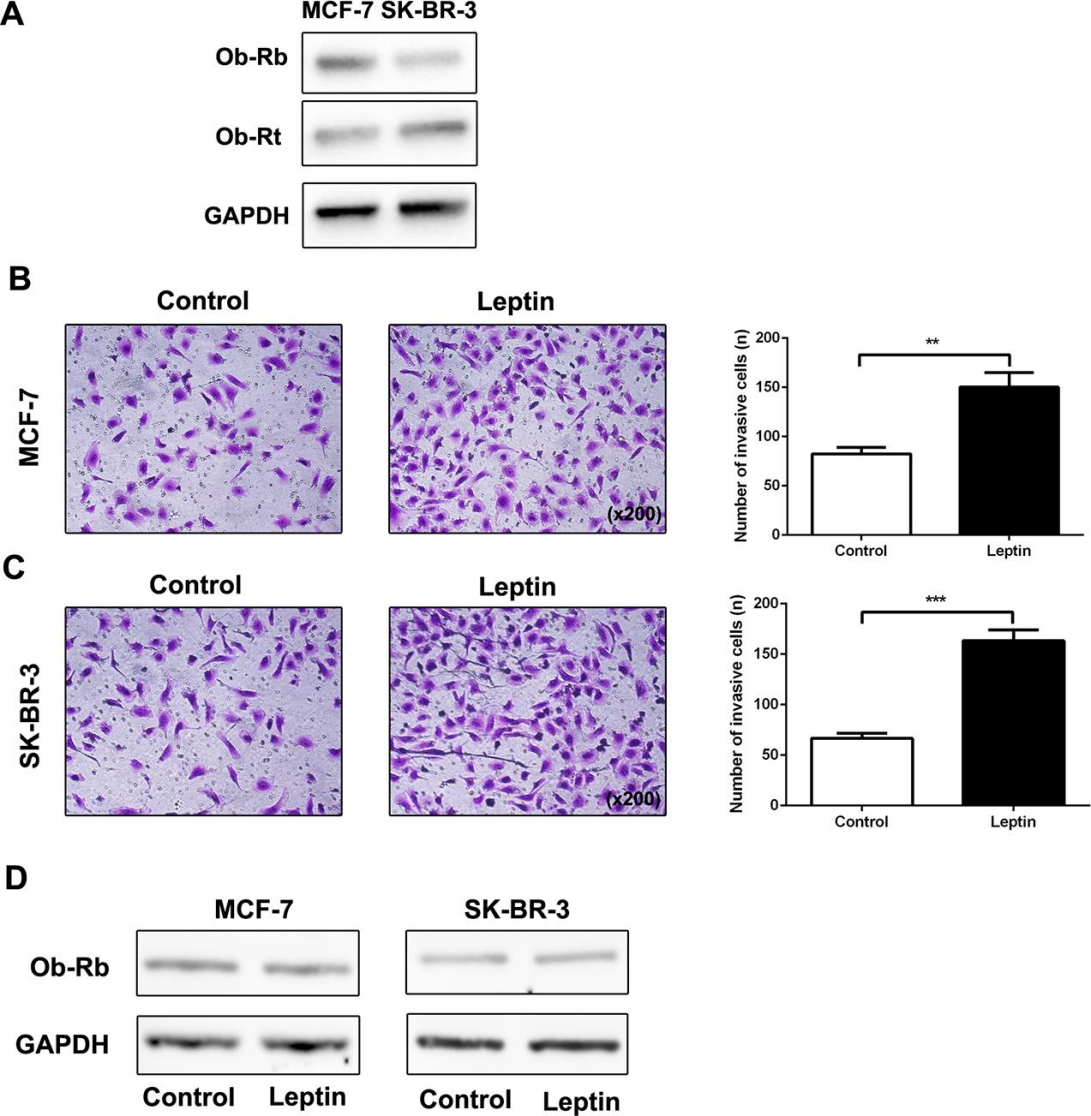
**Leptin promoted breast cancer cell metastasis and leptin receptor expression.** (**A**) Western blot was used to detect Ob-Rb and Ob-Rt expression in MCF-7 and SK-BR-3 cells. (**B**, **C**) The effect of leptin on invasion of MCF-7 and SK-BR-3 cells was examined using the transwell assay. The upper and lower chambers of the wells were separated by polycarbonate membranes. Breast cancer cells (5×10^4^/well) were seeded in the upper chambers of the 24-well plate with serum-free medium. The lower chamber was filled with medium containing 5% fetal bovine serum as a chemoattractant. The data are shown as means ± SD. **P<0.05. (**D**) Western blot was used to detect leptin receptor expression in MCF-7 and SK-BR-3 cells.

It has been reported that the biological function of leptin is mediated by specific Ob-R receptors [17]. We therefore examined expression of long (Ob-Rb) and short (Ob-Rt) leptin receptor isoforms in MCF-7 and SK-BR-3 cells. As shown in Figure 2A, expression of both Ob-Rb and Ob-Rt was observed in MCF-7 and SK-BR-3 cells. Next, we examined the effect of leptin (200 ng/mL) on metastatic potential in MCF-7 and SK-BR-3 breast cancer cells using a transwell assay. As shown in Figure 2B and 2C, leptin increased invasion of MCF-7 and SK-BR-3 cells compared to the control group. However, leptin did not upregulate leptin receptor expression in breast cancer cells (Figure 2D). These results indicate that leptin can effectively promote metastasis of breast cancer cells.

### Leptin receptor-mediated, leptin-induced epithelial-mesenchymal transition in breast cancer cells

**Figure 3:**
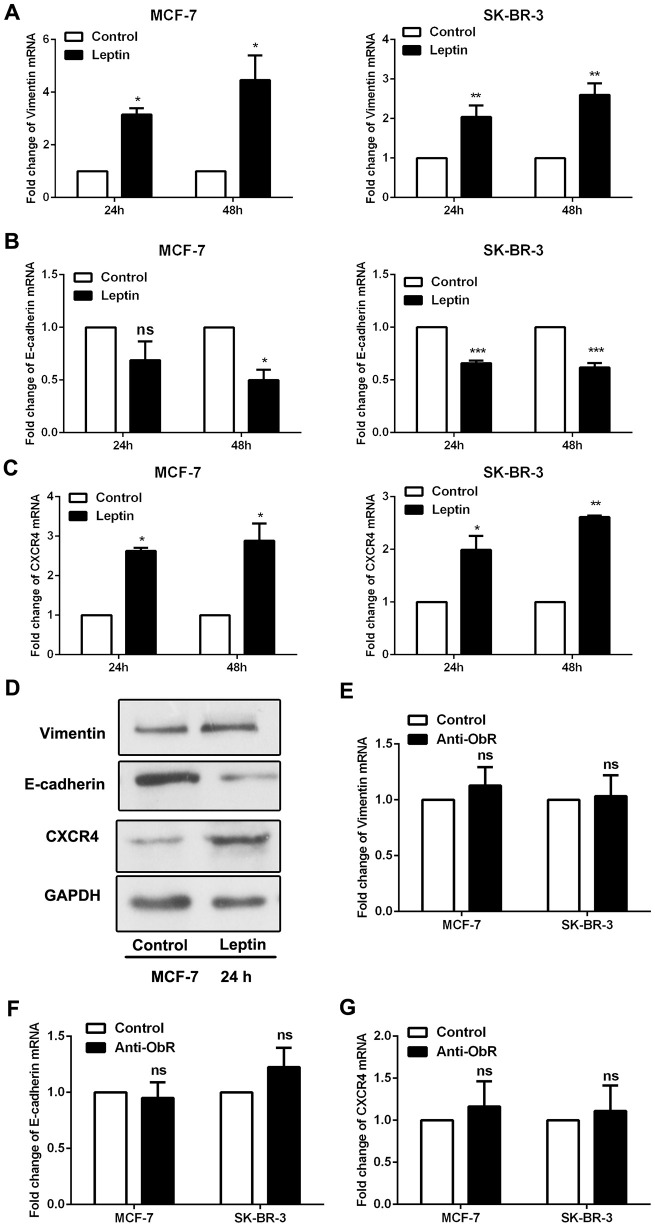
**Leptin receptor mediated, leptin-induced EMT in breast cancer cells.** (**A**–**C**) Real-time PCR was performed to examine Vimentin, E-cadherin, and CXCR4 expression in MCF-7 and SK-BR-3 cells. Data are shown as the means ± SD. *P<0.05, **P<0.01, ***P<0.001. (**D**) Western blot was employed to detect Vimentin, E-cadherin, and CXCR4 expression in MCF-7 cells. (**E**–**G**) Real-time PCR was used to detect Vimentin, E-cadherin, and CXCR4 expression in MCF-7 and SK-BR-3 cells. Data are shown as means ± SD. ns, not significant.

Epithelial-mesenchymal transition (EMT) is a crucial process in tumour metastasis [18]. The mesenchymal marker vimentin and epithelial marker E-cadherin were assessed to evaluate EMT in breast cancer cells. As shown in Figure 3A, 3B and 3D, leptin decreased E-cadherin expression and increased vimentin expression in breast cancer cells. CXCR4 was also recently reported as a marker of EMT [19]. We found that leptin also significantly increased CXCR4 expression in breast cancer cells (Figure 3C and 3D).

As shown in Figure 2, the ability of leptin to exert its effects depends on the expression of leptin receptors. We therefore used an ObR antibody to block leptin receptors and then observed the effects of leptin on EMT in breast cancer cells. As shown in Figure 3E–3G, leptin receptor inhibition significantly decreased the incidence of leptin-induced EMT. These results suggest that leptin induces EMT in breast cancer cells.

### Leptin-induced promotion of breast cancer cell metastasis involves the SDF-1/CXCR4 axis

**Figure 4:**
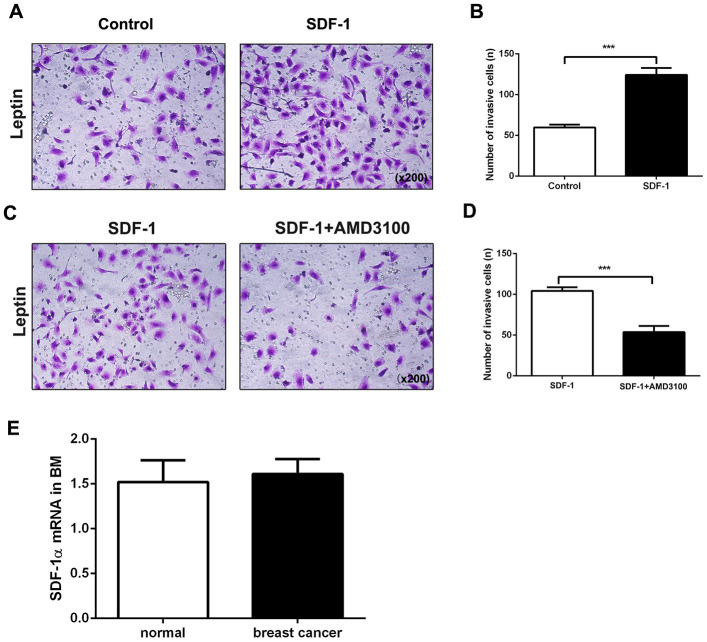
**The SDF-1/CXCR4 axis mediates leptin-induced promotion of breast cancer cell metastasis.** (**A**, **B**) Breast cancer cell invasion was examined using the transwell assay. The upper and lower chambers were separated by polycarbonate membranes. Breast cancer cells were pre-treated with leptin (200ng/mL) and then seeded in the upper chambers of the 24-well plate (2×10^4^/well) with serum-free medium. The lower chamber was filled with medium containing SDF-1 (100 ng/mL) as a chemoattractant. The data are shown as means ± SD. ***P<0.001. (**C**, **D**) The effect of the SDF-1/CXCR4 axis inhibitor AMD3100 on breast cancer cell invasion was examined in the transwell assay. Breast cancer cells were pre-treated with leptin (200 ng/mL) and then seeded in the upper chambers of a 24-well plate (2×10^4^/well) with serum-free medium containing AMD3100 (1 μg/mL). The lower chambers were filled with medium containing SDF-1 (100 ng/mL) as a chemoattractant. The data are shown as means ± SD. ***P<0.001. (**E**) Real-time PCR was used to detect SDF-1 expression in the bone marrow of healthy individuals and breast cancer patients.

Chemokine stromal cell-derived factor 1 (SDF-1) binds to chemokine (C-X-C motif) receptor 4 (CXCR4), which plays a vital role in tumor cell metastasis [20]. We therefore explored the role of the SDF-1/CXCR4 axis in leptin-mediated bone metastasis of breast cancer cells. Compared to the control group, coadministration of SDF-1 significantly increased leptin-induced invasiveness in breast cancer cells (Figure 4A, 4B). AMD3100, a CXCR4 inhibitor, was then used to examine the role of CXCR4 in breast cancer cell migration and invasion. Compared to the control group, AMD3100 effectively decreased metastasis in leptin-treated breast cancer cells (Figure 4C, 4D).

Having confirmed the important role of the SDF-1/CXCR4 axis in directional chemotaxis of breast cancer cells from breast to bone tissues, we performed real-time PCR assays to analyze SDF-1 expression in human bone marrow. As shown in Figure 4E, SDF-1 was constitutively expressed not only in the bone marrow of breast cancer patients, but also in normal bone marrow, indicating that SDF-1 might induce metastasis of CXCR4-positive breast cancer cells to bone tissues. Taken together, the above results suggest that leptin induces CXCR4 expression and activates the SDF-1/CXCR4 axis to promote metastasis of breast cancer cells to bone tissue.

### High CXCR4 expression promotes bone metastasis of breast cancer

**Figure 5:**
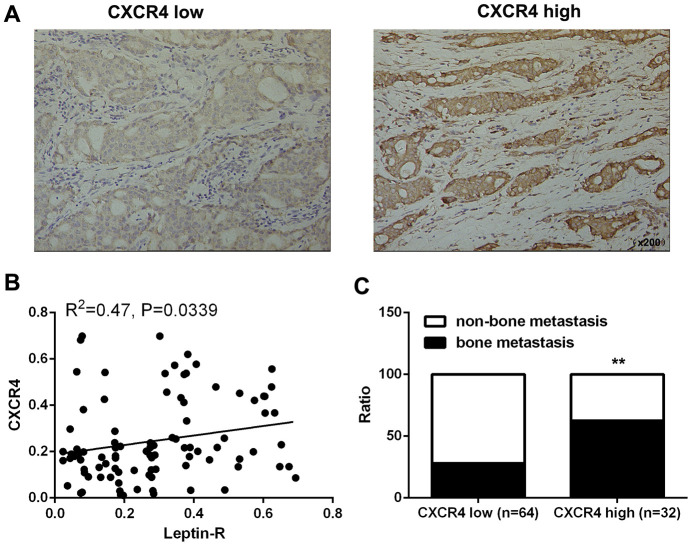
**High CXCR4 expression promoted bone metastasis of breast cancer.** (**A**) IHC staining was employed to examine CXCR4 expression in breast cancer tissues. (**B**) Integrated optical density (IOD) analysis indicated that Leptin receptor and CXCR4 expression were positively correlated in breast cancer patients. Pearson’s correlation analysis was used to determine the correlation coefficient (r) and p value. (**C**) Frequencies of bone metastasis and non-bone metastasis in patients with low or high CXCR4 expression.

In order to further understand the relationship between CXCR4 expression and bone metastasis of breast cancer, we examined the association between CXCR4 expression and clinicopathological features in breast cancer patients. Tissues were divided between two groups based on CXCR4 expression (high expression group, n=64; low expression group, n=32; Figure 5A). CXCR4 expression was positively correlated with leptin receptor levels (Figure 5B), and incidence of bone metastasis was higher (62.5%) in the CXCR4 high expression group than in the CXCR4 low expression group (28.12%, Figure 5C and Table 2). These results demonstrate that CXCR4 expression is associated with leptin receptor expression and bone metastasis in breast cancer patients.

**Table 2:** Correlation between CXCR4 expression and bone metastasis.

	**CXCR4 low (n=64)**	**CXCR4 high (n=32)**	**P value**
**non-one metastasis**	46	12	**0.001
**bone metastasis**	18	20	

** P<0.01

## DISCUSSION

Leptin, a known risk factor in several kind of cancers, promotes tumor cell development [21, 22]. The results of this study demonstrate that increased leptin expression promoted bone metastasis of breast cancer cells. Moreover, leptin-induced breast cancer cell metastasis was due at least in part to activation of the SDF-1/CXCR4 axis. High CXCR4 expression was associated with increased bone metastasis and poorer prognosis in breast cancer patients.

Although leptin receptor antagonist therapies are still undergoing clinical trials, leptin is still considered a candidate cancer treatment due to its unique status as a link between obesity and cancer [23, 24]. In addition, leptin can promote recurrence and distant metastasis in breast cancer, ultimately leading to poor overall survival in late stage breast cancer patients [25]. In agreement with these findings, we found that high leptin receptor expression in breast cancer tissue promoted bone metastasis and was associated with poorer overall survival. Our results therefore suggest that leptin might be an effective target in breast cancer therapy.

Epithelial-mesenchymal transition (EMT), a process associated with more malignant cell phenotypes, is closely related to tumor metastasis [26]. In EMT, immotile epithelial cells acquire mesenchymal characteristics and lose cell polarity and cell-to-cell adhesion, thus enabling increased migration and invasiveness [27]. Multiple factors in tumor tissue, including leptin expression, can promote aggressive phenotypes and tumor metastasis by promoting EMT. Xu et al. showed that leptin could induce EMT, and thus increase invasion and metastasis, by activating the ERK pathway in A549 lung cancer cells [28]. Gao et al. found that leptin-induced EMT can also promote peritoneal metastasis of ovarian cancer via the PI3K/Akt/mTOR pathway [29]. In this study, we found that leptin downregulated expression of the epithelial marker E-cadherin and upregulated expression of the mesenchymal marker vimentin in breast cancer cells. Furthermore, inhibition of leptin receptors in breast cancer cells significantly reduced the incidence of leptin-induced EMT. Thus, leptin induced the EMT phenotype in breast cancer cells.

Many previous studies have demonstrated the importance of the SDF-1/CXCR4 axis in tumor dissemination and cancer progression [11, 26, 30]. CXCR4 overexpression in tumor tissue is associated with poorer outcomes in several cancers, including breast cancer [31]. Several studies found that the SDF-1/CXCR4 axis played an important role in regulating the metastasis of breast cancer to specific organs [32–34]. Given this dichotomy between the physiological and pathophysiological functions of CXCR4, we examined CXCR4 expression in breast cancer cells and found that it was upregulated by leptin. We then examined the effect of SDF-1 on metastatic potential in breast cancer cells *in* *vitro* and found that it enhanced their metastatic potential. Additionally, leptin-induced metastasis of breast cancer cells was inhibited when the SDF-1/CXCR4 axis was blocked by AMD3100. These results suggest that SDF-1/CXCR4 signalling is activated by leptin and promotes metastatic features in breast cancer cells.

In conclusion, we demonstrate in this study that leptin was an important promoter of bone metastasis and influenced prognosis in breast cancer patients. Leptin induced the metastasis of breast cancer cells by activating the SDF-1/CXCR4 axis, and upregulation of CXCR4 contributed to bone metastasis and poor survival in breast cancer patients. These data suggest that treatments targeting leptin and CXCR4 in breast cancer warrant further investigation.

## MATERIALS AND METHODS

### Patients and samples

Ninety-six breast cancer patients who underwent treatment at the Tumor Hospital of Guangxi Medical University from 2009-2013 were included in this study. Patient followed-ups were performed every three months after resection. Clinicopathological features, including age, tumour grade, tumour size, and bone metastasis, were obtained from patient records. This study was approved by the Ethics Committee of Guangxi Medical University and all patients involved provided informed consent.

### Immunohistochemistry staining

Immunohistochemistry (IHC) staining was performed as described previously [35]. Deparaffinized sections (5 μm thick) were cut from array blocks and separately stained on an Autostainer Universal Staining System (LabVision, USA). For analysis, IHC staining levels were scored on a scale from 0 to 4 (0: no staining; 1: ≤10%; 2: 11% to 30%; 3: 31% to 50%; 4: ≥50%). Patients were divided between low (0, 1, 2) and high (3, 4) leptin receptor and CXCR4 expression groups based on IHC staining scores. For correlation analysis, IOD was evaluated in each image using Image-Pro Plus, Version 6.2 software (Media Cybernetics Inc., Bethesda, MD, USA); the expression value for each marker was calculated as IOD/total area of each image.

### Cell culture

MCF-7 and SK-BR-3 human breast cancer cells were cultured in RPMI 1640 medium (Gibco, USA) containing 10% fetal bovine serum (FBS, HyClone, USA) at 37°C in a 5% CO_2_ humidified atmosphere, and the medium was changed every three days. Cells from passages three to six were used in experiments.

### Wound healing assay

For the wound healing assay, 1× 10^5^ cells were seeded in 6-well plates and incubated for 24 h, after which the cell monolayers were scratched with sterile 10 μL micro pipette tips. Cell migration was quantified 24 hours after scratching using a phase-contrast microscope.

### Transwell invasion assays

Transwell invasion assays were performed using the Transwell system (Millipore, USA). The upper and lower chambers were separated by polycarbonate membranes. Breast cancer cells were seeded in the upper chambers of a 24-well plate with serum-free medium. The lower chamber was filled with medium containing SDF-1 (100 ng/mL) or 5% fetal bovine serum as chemoattractants. The 24-well plate was cultured at 37°C in a 5% CO2 humidified incubator for 24 hours, and cells remaining in the upper chamber above the filter membrane were removed. Cells on the bottom of the membrane that had infiltrated the lower chamber were fixed with paraformaldehyde (4%) and stained with crystal violet (0.1%) for 10 minutes. Finally, the stained cells were counted using a microscope (100×) (Olympus, Japan). The average number of cells that had passed through the artificial basement membrane in five random fields was used as the measure of cell invasiveness.

### Realtime-PCR

Total RNA was isolated from cells using Trizol reagent (TAKARA, Japan) according to the manufacturer’s instructions. cDNA was then generated using the PrimeScript RT reagent kit (Takara, Kyoto, Japan). CXCR4, SDF-1, E-cadherin, and Vimentin mRNA expression were quantified by real-time quantitative PCR using the SYBR Green PCR Kit (Applied BI). Thermal cycler conditions included two initial holds at 50°C for 2 minutes and 95°C for 10 minutes followed by a two-step PCR program of 15 seconds at 95°C followed by 1 minute at 60°C repeated for 40 cycles on an Mx4000 system (Stratagene, La Jolla, CA), on which data were collected and quantitatively analyzed. mRNA expression levels are presented as fold change relative to an untreated control. The sequences of the PCR primers are shown in Table 3.

**Table 3:** The sequences of PCR primers.

**Gene**		**Sequence (5′–3′)**
E-cadherin	F	CGAGAGCTACACGTTCACGG
	R	GGGTGTCGAGGGAAAAATAGG
Vimentin	F	GCCCTAGACGAACTGGGTC
	R	GGCTGCAACTGCCTAATGAG
CXCR4	F	ACTACACCGAGGAAATGGGCT
	R	CCCACAATGCCAGTTAAGAAGA
SDF-1	F	GCACTCGCACGACATCAAATA
	R	CTTGCCCGTAAGCACATGC
GAPDH	F	TGTGGGCATCAATGGATTTGG
	R	ACACCATGTATTCCGGGTCAAT

### Western blot

Total protein was extracted from tissues and stored at -80°C degrees until further analysis. SDS-PAGE gels were used to separate proteins by polyacrylamide gel electrophoresis, after which the proteins were transferred to PVDF membranes and then blocked with 5× protein gel electrophoresis loading buffer. Membranes were incubated with primary antibody overnight at 4°C (ObR (1:200, Santa Cruz, USA); E-cadherin (1:1000, Thermo fisher, USA); vimentin (1: 1000, GeneTex, USA); CXCR4 (1: 1000, LifeSpan BioSciences, USA)) and then with corresponding secondary antibodies at room temperature for 1 hour. Antibody binding was visualized with a UVP gel imaging analysis system, and LabWorks analysis software was used to analyze the gray values.

### Statistical analysis

All the experiments were performed at least three times independently (n ≥ 3). GraphPad Prism 5.0 (GraphPad Software) was used for statistical analysis. Quantitative data are expressed as mean ± SD. Student’s t-test was used to assess the significance between groups. Statistical significance is indicated as follows: *P < 0.05; **P < 0.01; ***P < 0.001.
